# Research Methods in a Multinational Business Environment and Implications for Capital Formation: Application of Cross-Sectional Autoregressive Distributed Lag Methods

**DOI:** 10.3389/fpsyg.2022.867891

**Published:** 2022-06-01

**Authors:** Musaad M. Halwan, Zhang Y. Bin, Waqar Ameer, Nosheen Mumtaz, Ayesha Mumtaz, Azka Amin

**Affiliations:** ^1^School of Economics and Trade, Hunan University, Changsha, China; ^2^School of Economics and Trade, Shandong Technology and Business University, Yantai, China; ^3^School of Economics and Management, Anhui University of Science and Technology, Huainan, China; ^4^School of Public Administration, Hangzhou Normal University, Hangzhou, China; ^5^Department of Business Administration, Sukkur Institute of Business Administration University, Sukkur, Pakistan

**Keywords:** foreign direct investment, institutional quality, domestic capital formation, cross-sectional dependency, panel data

## Abstract

We explore whether foreign direct investment outflows augment or obstruct public or private capital in developing countries by decomposing domestic capital into private and public capital. While developed countries are the primary source of foreign direct investment outflows (FDIOs), developing economies have become the primary source of FDIO over the past 30 years. We apply cross-sectional autoregressive distributed lag (CS-ARDL) methods to overcome the issue of endogeneity and cross-sectional dependency in our dataset. This study analyzes the interaction effects of foreign direct investment and institutional quality (IQ) in promoting aggregate domestic capital formation in developing countries. Our empirical results show that FDI outflows augment private capital formation and additionally, IQ also upsurges private capital formation. Conversely, as per results, FDI outflows obstruct public capital formation, and IQ crowds out public capital formation significantly while private capital crowds out FDI inflows. As per result estimations, we notice that FDIO crowds in private capital formation, thus we conclude that the private sector controls the majority of the sectors for developing countries and the role of the public sector is quite minimal. We conclude that private and public capital possess different attributes; thus clubbing them together might result in aggregation bias. Our result estimations provide several useful policy implications.

## Introduction

Foreign direct investment inflow (FDII) is the crucial channel of technology transfer from industrialized to developing countries (Ali et al., 2021; Chandio et al., 2021; Abbasi et al., 2022), and thus it contributes significantly toward the economic development of developing countries (Hao et al., 2021; Irfan et al., 2021; Tanveer et al., 2021). FDI inflows have rapidly increased in the last two decades particularly in developing countries (Irfan and Ahmad, 2021; Khan et al., 2021; Rauf et al., 2021). Foreign direct investment (FDI) fulfills rising investment requirements to boost economic growth in developing countries (Razzaq et al., 2021; Wu et al., 2021; Tang et al., 2022). Even though developed countries are the principal source of foreign direct investment outflows (FDIOs), developing economies have emerged as the leading sources of FDIO in the last three decades (Knoerich, 2017; Amin et al., 2020; Xiang et al., 2022). Several multinational companies from developing and emerging countries are investing across the borders globally through FDI. From 1980 to 2011, the developing countries’ share of world FDIO increased from 6.2 to 26.9% and rose to the highest level in 2010 by 31.8% (Al-sadiq, 2013). Thus, there is an interesting question whether FDIO significantly affects domestic capital formation (DCF) or economic activities in developing or emerging economies.

**FIGURE 1:**
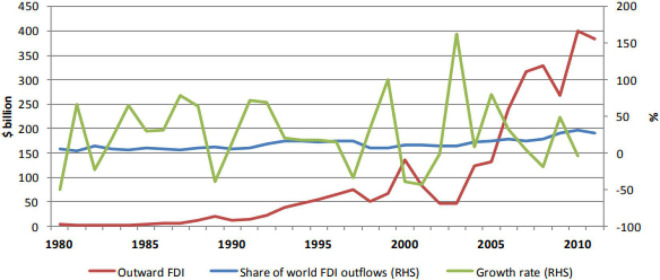
Trend of Outward FDI in Developing Economies from 1980 to 2011(UNCTAD, 2012; Al-sadiq, 2013).

While the rising trend of FDIO from developed countries is seriously discussed and empirically analyzed in the existing literature to investigate the impact of capital outflows on their domestic capital formation, it raises serious questions why no attention is paid to analyze such incoming effects of FDIO on aggregated DCF particularly in developing countries. Policy advisers need to complete in-depth analysis to accurately determine how domestic capital formation is affected by FDI outflows in developing countries. Domestic capital investment determines the physical capital accumulation rate which determines the economic growth rate afterward. Comprehending the mechanism of capital outflows on domestic capital formation is a prerequisite step toward introducing market reforms that can augment domestic capital formation and accelerate economic growth (Al-sadiq, 2013; Moraru, 2013; Nistor, 2014; Mirela et al., 2015). Figure 1 clearly indicates that the share of developing countries in world FDIO rose consistently from 1980 to 2011 annually. In 1982, developing countries’ share of world FDIO rose at a peak level of 9.6%; the share of developing countries of world FDIO further increased in 1994 by 16.7%. This share declined sharply between 1998 and 2001 but subsequently rose to the peak level of 31.8 in 2010 (Al-sadiq, 2013).

Domestic capital plays a key role in the economic development and diversification of a country (IMF, 2018)^1^ and it works in the role of catalyst to mitigate the repercussions of economic shocks and upsurge economic activities to stabilize the economy in the home country. FDII and FDIO can follow different channels to stimulate domestic capital. FDI inflows augment domestic investment by importing capital and advance technology along with technical knowledge in the home country (Al-Iriani, 2007; Ghassan and Alhajhoj, 2016), while FDI outflows expand domestic investment by interconnecting local investors to the global chain of business in the multinational corporate environment (Desai et al., 2005; Al-sadiq, 2013). Compared to FDI inflows, the incoming effects of FDI outflows on domestic capital are complicated. FDI outflows affect domestic capital through two channels (Stevens and Lipsey, 1992). Firstly, FDI outflows stimulate domestic capital if financed through abundant capital and excessive foreign reserves of the home country, known as “fire power” by Wu (2008). If home countries are not abundant in domestic savings, FDIO financing can tremendously decrease the domestic capital of the home country. Secondly, FDIO financing can reduce the cost of production and significant raise returns to domestic capital of the home country by utilizing efficient capital and cheap labor abroad, thus paving the way for the expansion of domestic capital in the home country (Desai et al., 2005). However, the outcome of these two channels depends mainly on the motives of FDIO (Hejazi and Pauly, 2005; Al-sadiq, 2013).

Institutional quality (IQ) promotes economic development. Institutional quality is supposed to reduce the uncertainty associated with exchange, and thus, it provides a solid platform for successful completion of legal economic transactions by reducing the likelihood of default risk. Parties at the opposite ends have insufficient information about their counter partners’ true business intentions which might cheat or defraud them to earn optimal business profits. Owing to uncertainty linked with business transactions, risk premium is included in the transaction costs. Risk premium depends on the enforceability of the contract, protection of property rights, and probability of the default risk, and thus, it is the function of institutional quality. Lower investment, lesser productivity growth, and lower per capita income is highly linked with poorer institutional quality. Hence, this slow process causes overall slower economic growth (Jude and Levieuge, 2013). Moreover, developing economies have high returns for strong institutional quality relative to those of developed countries.

The classical theory claims that private and public capital formation typically crowds out each other (Şen and Kaya, 2014; Mohanty, 2018). Literature regards the attainment of private and public investment very differently (Narayan, 2004; Şen and Kaya, 2014; Rehman, 2016; Mohanty, 2018; Sultanuzzaman et al., 2018; Shah et al., 2019). In general, private and public capital formations repel and resist each other (Şen and Kaya, 2014; Mohanty, 2018). Consequently, accumulating them into one composite term may cause domestic capital formation aggregation bias. The main question that we discuss in this empirical study is the decomposition of aggregate capital formation into private and public capital with particular focus on developing countries. Empirical literature considers that public and private capital are quite different (Narayan, 2004; Shah et al., 2019). Private capital formation enhances productivity, encourages diversity, and increases efficiency in the economy while public capital is considered to have the opposite effect. Hence, overall, such types of capital, i.e., public and private, resist each other and aggregating them together into one composite term may create the problem of aggregation bias.

Several studies, such as Buchanan et al. (2012) and Huynh et al. (2020), explored the impact of inward FDI on domestic investment at the macro-economic level for developing countries but these studies did not consider the idea of decomposition of domestic investment. Also, a recent study of Amighini et al. (2017) explored the nexus between domestic capital formation and FDI inflows at the industry level, but this study did not consider the idea of decomposition of DCF, i.e., private and public capital. In addition, these studies also ignored the issue of common correlation bias which generally exists in panel time-series data. Studies of Shah et al. (2020) and Ameer et al. (2021) considered the idea of bifurcation by decomposing aggregation capital formation into private and public capital with particular focus on GCC regions but not especially developing regions. To the best of our knowledge, there is only a single study by Morrisey and Udomkerdmongkol (2012) which has explored the impact of FDI inflows and institutional quality on private investment with particular focus on the developing region by decomposing aggregate capital formation into private and public capital formation.

Although the study of Morrisey and Udomkerdmongkol (2012) provides valuable insight by exploring the nexus between governance, FDI inflows, and domestic capital, particularly for developing countries by bifurcation of domestic capital into private and capital formation, this study also suffers from significant shortcomings such as missing notable key variables, i.e., FDIO and interactive proxy variables. Additionally, this study just explored the impact of FDI inflows and governance on private capital formation but ignored the impact of FDI inflows on public capital. As per statistics (see Figure 1 and Table 1), we notice that the developing countries’ share of world FDIO rose at a peak level in the last couple of decades. Accordingly, it is pretty interesting to consider the role of FDIO in our proposed study. Henceforth, to avoid the omitted variable or model misspecification bias and provide more robust checks to the existing empirical studies, we have added key notable variables in our model. The study by Morrisey and Udomkerdmongkol (2012) applied Generalized Method of Moments (GMM) methods to control endogeneity issues, still it does not overcome the problem of common correlation bias, which generally exists in panel data studies. Henceforth, this study controls the issue of endogeneity and cross-sectional dependency issues by applying advanced methods of cross-sectional autoregressive distributed lag (CS-ARDL).

**TABLE 1:** Outward FDI by Region: 1980–2011.

Region	1980s	1990s	2000-2005*	2006	2007	2008	2009	2010	2011
World	93.5	418.8	814.6	1415.1	2198.0	1969.3	1175.1	1451.4	1694.4
Advanced countries	87.6	372.7	711.7	1152.0	1829.6	1580.8	857.8	989.6	1237.5
Developing Economies	5.9	44.9	94.6	239.3	316.9	328.1	268.5	400.1	383.8
Africa	0.5	1.9	0.8	8.2	9.3	7.9	3.2	7.0	3.5
Latin America	1.1	9.9	32.2	79.7	79.3	97.0	54.3	119.9	99.7
Asia and Oceania	43	33.1	61.6	151.4	228.2	223.2	211.0	273.2	280.6
MENA	0.69	0.08	3.9	23.0	37.8	44.4	19.2	20.0	24.9
Transition Economies	0.0	1.2	8.3	23.7	516	60.5	48.8	61.6	73.1

Billions of current U.S. dollars. *Annual average.

This is the first study, particularly for developing countries, which has addressed the issue of aggregation bias by comprehensively exploring the simultaneous impact of FDIO, FDII, and IQ on aggregate domestic capital by decomposing aggregate capital formation into private and public capital formation separately. Thus, we extend the contribution of this proposed study by exploring the simultaneous impact of FDII, FDIO, and IQ on private and public capital, respectively. Next, we explore the interaction effects (FDIO*FDII*IQ) of FDII, FDIO, and IQ in stimulating private and public capital formation separately by bifurcating the domestic capital formation in private and public capital to avoid the issue of avoiding aggregation domestic capital formation bias. We expect a positive impact of FDIO, FDII, and IQ on DCF which is a good sign for developing countries. Ignoring the cross-sectional dependency issue in panel time series data may result in biased estimation, therefore this paper uses the novel concept of CS-ARDL to determine cross sectional dependence and endogeneity issues in the developing region and get consistent results.

### Overview of the Trend of Foreign Direct Investment Outflows From a Developing Countries Perspective

**FIGURE 2:**
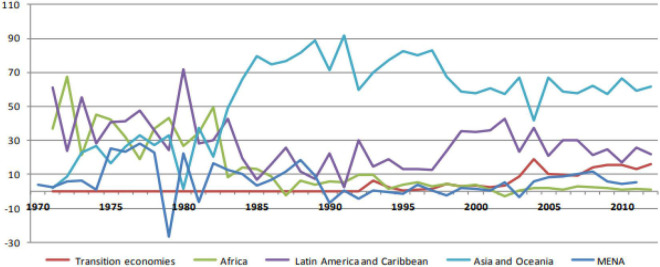
Trend of Rise of FDIO in Asia, Africa, and Latin America (UNCTAD, 2012).

As per Figure 2 and Table 1, we notice that FDIO from developing countries has increased significantly in the last three decades. According to Table 1, 61.4% of the total FDIO from developing countries originated from Asia, 21.8% of total FDIO originated from Latin America and the Caribbean, and 16% of the total FDIO originated from transition economies (IMF, 2013).

Since 1985, Asian countries have been the largest source countries and top destinations among developing economies for originating FDIO to other host countries. Alternatively, a rise of FDIO from Africa was much less comparative to other developing regions. On the contrary, FDIO that originated from the African region is quite minimal compared with those of other developing countries.

Our proposed empirical study is categorized into different sections. The second section comprises the theoretical and empirical literature review. The third section comprises data sources and methodology. The results and discussions are presented in the fourth section. Finally, the fifth section is based on the conclusion and provides interesting policy implications based on empirical estimations.

## Literature Review

### Theoretical Literature

The incoming effects of FDIO on domestic capital formation differ from one country to another, depending on the firms’ key objectives to invest abroad and the home country’s economic condition. Stevens and Lipsey (1992) discuss different channels by which capital outflows affect the home country’s domestic capital formation. The first channel through which FDIO affects the home country’s domestic capital is through financial markets. Firms that plan to invest abroad will transfer some portion of their domestic savings in the host country, thus some portions of the domestic capital will be transferred abroad to the host country. Hence, under the markets of scarcity of domestic sources in the home country, the existing financial resource to invest in new projects will be curtailed and domestic firms will have to face financial constraints while raising funds for investment projects. Thus, based on this mechanism, FDIO decreases domestic capital particularly if firms finance external foreign investment by internal capital. The second channel through which FDIO affects a home country’s domestic capital is through product markets by shifting their production abroad. The literature regards three motives of FDIO through shifting production abroad. Hence, these three motives of investing abroad are efficiency-seeking, market-seeking, and strategic asset–seeking (Dunning, 1993).

The efficiency-seeking FDIO motive occurs when firms want to increase its efficiency by transferring their production abroad to the host country with relatively cheap labor and lesser costly raw material and inputs; it is also called vertical FDI (Hejazi and Pauly, 2005). When firms export capital and intermediate goods, no initial reduction is expected in the domestic capital and thus, FDIO will expand domestic capital in this scenario (Hejazi and Pauly, 2005). Depending on whether FDIO displaces the exports, its incoming effects on domestic capital will remain neutral. On the other hand, while displacing the exports of final products, it may boost the exports of intermediate goods from the parent or other firms in the home country; the net impact remains unclear (Hejazi and Pauly, 2005). The second FDIO motive focuses on serving the host country’s domestic and neighboring markets and the effects of market-seeking FDI are quite unclear. Thus, under these conditions it depends whether FDIO replaces or displaces exports (Hejazi and Pauly, 2005). The strategic asset–seeking motive of FDIO aims to obtain assets which are unavailable in the home country that might be quite important for the firms’ long term sustainable development. This type of FDIO may have favorable effects on domestic capital due to access to advance technology and knowledge, thus it helps firms increase their efficiency and productivity in the home country. In sum, the impact of FDIO on domestic capital may vary from one country to another as per the economic structure of the home country. Hence, the incoming effects of FDIO on domestic investment might be neutral, positive, or negative, depending on the economic characteristics of the home country (Al-sadiq, 2013).

### Empirical Literature

Generally, the empirical literature on the relationship between FDIO and domestic capital is divided into two strands. The first strand uses firm–level data while the other strand works on the aggregate level at a macro-level perspective. The results provided by both strands are indecisive. A few studies found that a home country’s domestic capital is reduced with FDIO while other research found that FDIO stimulates domestic capital, and many found no effect (Desai et al., 2005; Hejazi and Pauly, 2005; Al-sadiq, 2013). Based on empirical evidence of OECD countries, Feldstein (1995) finds that one dollar increase in FDIO reduces domestic investment by one dollar. Feldstein (1994) and Desai et al. (2005) conducted analysis at a macroeconomic level and concluded that FDIO reduces domestic capital in a one-to-one ratio. Andersen and Hainaut (1998) explored the nexus between FDIO and domestic investment from the 1960s to 1990s annually. This study particularly focused on Germany, Japan, United Kingdom, and United States and its result findings strongly support the empirical estimations of Feldstein (1995) and Desai et al. (2005) that FDIO reduces domestic investment by an approximately one-to-one ratio.

Based on the empirical evidence of seven multinational enterprises in the US for the time span of 20 years, Stevens and Lipsey (1992) found a strong positive connection between FDI outflows and domestic capital. A study of Herzer and Schrooten (2007) concluded that FDIO reduces domestic investment in the long run for Germany while incoming effects of FDIO on domestic investment are positive in the short run. However, for the United States, FDIO expands domestic investment significantly in the long run but it decreases domestic investment in the short run. Sunesen et al. (2010) found that FDI outflow helps to increase the production of domestic firms. While, Herzer (2010, 2008) found that FDIO plays a key role in promoting economic growth and development of the home country. For Malaysia, Chen and Zulkifli (2012) investigated the relationship between FDIO and economic growth by employing the vector error-correction model. Additionally, You and Solomon (2015) conducted analysis with industrial-level data and found that FDIO expands domestic investment by applying GMM. Knoerich (2017) found FDIO positively contributes in the economic development of an economy. Ameer et al. (2017) explored the nexus between FDIO and domestic capital formation particularly in the case of China and concluded that there exists a unidirectional positive long-run causality running from FDIO to aggregate domestic capital, however, a causality relationship does not exist in the short run. While, the results of Amin et al. (2020) demonstrate that FDIO increases domestic output as well as overseas production to stimulate their competitiveness worldwide. Neoclassical theories consider that FDI is the crucial factor in the stimulation of domestic capital reserves in addition to the expansion in the total factor productivity^2^ (De Mello, 1997; Xu and Wang, 2007). Modernization hypotheses claim that FDI inflows play a significant role in the expansion of domestic capital, technology transfer, market liberalization, and modernization of infrastructure in addition to increases in labor productivity and upgradation in managerial policies (Kumar and Pradhan, 2002). Conversely, several research studies conclude that FDI inflows decrease domestic capital in the long run (Braunstein and Epstein, 2002).

After thoroughly reading the empirical literature, we notice that studies of Buchanan et al. (2012) and Huynh et al. (2020) have explored the impact of aggregate domestic capital on FDI inflows for developed or developing countries but these studies did not consider the idea of the decomposition of aggregate domestic capital formation. Moreover, few existing empirical studies, such as Shah et al. (2020) and Ameer et al. (2021), investigated the impact of FDI outflows and inflows simultaneously on DCF by bifurcation of aggregate capital formation into private and public capital and these two studies particularly focused on the GCC region but not developing regions. Henceforth, only the study of Morrisey and Udomkerdmongkol (2012) has investigated the impact of FDI inflows and institutional quality on private capital formation with particular focus on the developing region by decomposing aggregate capital formation into private and public capital formation. After reading the relevant literature, we extend the contribution of this proposed study by exploring the simultaneous impact of FDII, FDIO, and IQ on private and public capital, respectively, particularly with focus on developing countries. This paper aims at specifically filling this gap.

## Data and Methodology

Our empirical research study comprises of a panel of 61 developing economies over the time span from 1998 to 2017 annually. We have a large panel of countries *(N > 61)* and a relatively smaller time span *(T ≤ 20)* in our proposed study. Hence, it is highly expected that the FDIO of one developing economy will influence the FDIO of other developing economies when we have a large number of cross-country studies (N) and a relatively smaller number of years (T) due to the effect of globalization and technology advancement. Also, FDI inflows or institutional quality of one developing country may also influence FDI inflows or institutional quality of other developing countries due to the rising wave of globalization, regional policies, and technology advancement. Henceforth, the possibility of cross-sectional dependence cannot be ruled out in the era of globalization and technology advancement particularly when developing countries closely collaborate for their regional cooperation, economic prosperity, and development with particular focus on developing regions. Common correlation bias generally arises due to spill-over effects, omission of common factors, and intragroup interactions within similar socioeconomic networks (Pesaran and Tosetti, 2011). Accordingly, the possibility of cross-sectional dependency (CD) is highly expected in the case of developing countries. To check for the cross-sectional dependency issue, we formulate our null hypothesis that variables of our study are cross-sectionally independent while the alternative hypothesis considers that variables of our study are cross-sectionally dependent. Thus, this empirical work applies the methods of Pesaran (2004).


(1) $$\text{CD}={\left(\frac{\mathrm{TN}(N-1}{2}\right)}^{1/2}\,\bar{\hat{\rho }}$$

where $\bar{\hat{\rho }}=\left(\frac{2}{N\,\left(N-1\right)}\right)\,\sum_{\text{i}=1}^{N-1}\sum_{\text{j}=\text{i}+1}^{\text{N}}{\hat{\rho }}_{\text{ij}}$ and${\hat{\rho }}_{i\,j}$ denotes the pair-wise correlation coefficient of the residuals which is derived from ADF regression. Our baseline regression equation is as follows:


(2) DCF=itα+iβFDIOi1+itβFDIIi2+itβIQi3+itεit

DCF denotes aggregate domestic capital formation in equation (2). Our core variable of interest is FDIO, but we are also quite excited to know the impact of FDII and IQ on DCF for developing economies. To exactly explore the impact of FDIO, FDII, and IQ on DCF, we also add additional control variables in our model by extending our baseline model of equation (2). The selection of control variables in our proposed study is based on the existing literature relevant to this study’s topic and it is denoted by “x.” Where, *i* stands for the cross-sectional dimension *such as i* = *1…….i*, *t* stands for time period such as *t* = *1*…….*t*, and α_*i*_ represents country-specific effects. **α _*i*_** denotes the coefficient of the intercept term and **β _*it*_** signifies the respective coefficient of the relevant explanatory variable in our model of study such as **β _*i*1_ β _*i*1_ = α _*i*1_/*1*-α _*i*1_, β _*i*2_ = α _*i*2_/1-α _*i*1_**, *and* **β _*i*3_ = α _*i*3_/1-α _*i*1_**. The extended model of our proposed study is reported below in equation (3):


(3) DCF=itα+iβFDIOi1+itβFDIIi2+itβIQi3+itβxi4+εit

The error term and coefficients of our extended model in equation (3) follow similar attributes of our baseline model in equation (2). Henceforth, we proceed further by addition of control variables, such as the interaction variable (FDIO × FDII × IQ) and inflation rate, in our baseline model in equation (2) in order to more comprehensively analyze the impact of FDIO, FDII, and IQ on DCF in the developing region. The addition of control variables will not only provide robust checks to result findings but also provide the true overview of the economic condition of the developing countries. To go further in our in-depth analysis, we investigate the impact of exploring the incoming effects of FDIO, FDII, IQ, and other variables of interest on the public and private capital formation, respectively, in the developing countries. We will replace aggregate DCF in equations (2) and (3) with PRI (private capital formation) and PUBI (public capital formation) alternatively.

### Issue of Cross-Sectional Dependency and Panel Unit-Root Testing Methods

Panel time series data require stationarity checks for the variables of our proposed study. Modern research in the area of econometrics has reported new advanced techniques of panel unit root methods in order to produce unbiased result estimations. The traditional panel unit root test methods consider that there is no cross-sectional dependence across variables (Maddala and Wu, 1999; Levin et al., 2002; Im et al., 2003). Though, the modern panel unit root test methods not only control cross-sectional dependence across units but also overcome the issues and discrepancies of structural breaks in the panel time series data (Moon and Perron, 2004; Smith et al., 2004; Pesaran, 2007). In order to check stationarity among the variables of our proposed study, we apply cross-sectional augmented Dickey-Fuller (CADF) panel unit root methods as given below:


(4) △⁢yit=αi+K⁢tii+βi⁢yit-1+γi⁢y¯t-1+ϕi⁢Δ⁢y¯t+εit

Where i=1,.,N*and*t=1,.,T, and${\bar{y}}_{\text{t}}$ indicates the cross-sectional mean value of y_it_ and it is estimated from ${\bar{y}}_{\text{t}}={\text{N}}^{-1}\,\sum_{\text{i}=1}^{\text{N}}{\text{y}}_{\text{it}}$. In equation (3), the null hypothesis is H_0_:β_i_ = 0 (there is no cross-sectional dependency across the units) for all i and the alternative hypothesis is H_a_:β_i_0 (there is cross-sectional dependency across the units) for some i. The panel augmented unit root test of cross-sectional dependence (CIPS) is proposed by Pesaran (2007) and its test statistics are as follows:


(5) $$\text{CIPS}\,\left(N,T\right)={\text{N}}^{-1}\,\sum_{\text{i}=1}^{\text{N}}{\text{t}}_{\text{i}}\,\left(N,T\right)$$

*t*_*i*_(*N*,*T*) in equation (5) indicates the t-statistic forβ_*i*_. N and T stand for the number of cross-country units and total number of years, respectively. We have applied a cross-sectional dependency (CD) test in order to detect cross-sectional dependency across the units. Panel unit root (CIPS) and CD test results are displayed in Table 2. The results show that all the variables are stationary at the first difference and there exists cross-sectional dependency across all the variables of study. Accordingly, we apply CS-ARDL methods for this empirical study.


(6) $$\begin{array}{c} △\,{\text{Y}}_{\text{it}}=\mu_{\text{i}}+\varphi_{\text{i}}\,\left(Y_{\mathrm{it}-1}-\beta_{\text{i}}\,{\text{X}}_{\mathrm{it}-1}-\phi_{1\,i}\,{\bar{\text{Y}}}_{t-1}-\phi_{2\,i}\,{\bar{\text{X}}}_{t-1}\right)+\sum_{\text{j}=1}^{p-1}\lambda_{\text{ij}}\,\Delta \,{\text{Y}}_{\mathrm{it}-j}+\sum_{\text{j}=0}^{q-1}\zeta_{\text{ij}}\,\Delta \,{\text{X}}_{\mathrm{it}-j}+\eta_{1\,i}\,\Delta \,{\bar{\text{Y}}}_{\text{t}}+\eta_{2\,i}\,\Delta \,{\bar{\text{X}}}_{\text{t}}+\varepsilon_{\text{it}} \end{array}$$

**TABLE 2:** CD test and second-generation panel unit root.

Variable	ρ\^hfil CD	Levels CIPS	First differences CIPS	
FDIO	0.220	16.30***	−2.936***	−15.276***
IQ	0.441	15.88***	1.759	−9.807***
FDII	0.271	15.68***	–0.252	−16.554***
FDIO*FDII*IQ	0.210	2.89***	–0.897	−14.101***
PRI	0.487	37.33***	–0.553	−8.410***
PUBI	0.462	35.57***	1.075	−9.571***
INFLATION	0.249	20.39***	−8.090***	−18.89***

*, **, and *** denote the level of significance at 1, 5, and 10%, respectively.

*Y_*it*_ (DCF or PUBI or PRI)* are the dependent variables of our empirical study, *μ_*i*_* is the intercept value, and β_it_ is the slope coefficients of the explanatory variables and lagged dependent variables. ***X*_*it*_** (*FDIO, FDIO, and IQ*) is the vector of independent variables whereas φ_*i*_is the error correction term (ecm). The negative and significant value of φ_*i*_ (ecm) indicates the adjustment of short-run disequilibrium after abnormal economic shocks toward long-run equilibrium.${\bar{Y}}_{t-1}$ and ${\bar{X}}_{t-1}$ terms denote the unobserved factors in the long run while $\Delta \,{\bar{Y}}_{t}$and $\Delta \,{\bar{X}}_{t}$ indicate unobserved factors in the short run.

### Dataset Sources and Theoretical Justification

DCF or PRI or PUBI are the dependent variables in this empirical study while the rest of the variables are independent variables which are reported in Table 3. We have considered six individual governance indicators to denote institutional quality. These six individual governance indicators^3^ are GS, DA, LO, BQ, SE, and COR. We extracted PCA^4^ of six individual governance indicators and termed these aggregated composite terms as institutional quality and the correlation matrix of these individual governance indicators is displayed in Table 4.

**TABLE 3:** Description and source of the variables.

Variable	Description	Theoretical justification	Source
DCF	Domestic capital formation	Domestic capital formation enhances productivity, endorses diversity, and increases efficiency in the economy and thus, it contributes toward economy development in the country (Narayan, 2004).	World development indicators (WDI)
FDIO	Foreign direct investment outflow	The effects of FDIO on domestic investment depends on the firm’s motive to invest abroad and the home country’s economic conditions. Accordingly, FDIO effects on aggregate domestic capital incoming might be positive, negative, or neutral as its effects may vary on the firm’s purpose to invest abroad and the home country’s economic condition (Hejazi and Pauly, 2005; Al-sadiq, 2013).	WDI
FDI	Foreign direct investment inflow	FDI inflows fulfill rising investment needs of the developing countries and contribute toward the economic development of the developing states (Ndikumana and Verick, 2008).	WDI
PRI	Private capital formation	Private capital formation enhances productivity, endorses diversity, and increases efficiency in the economy while public capital is considered the other way around (Narayan, 2004; Şen and Kaya, 2014; Mohanty, 2018; Shah et al., 2019).	IMF fiscal affairs department
PUBI	Public capital formation	Literature regards the attainment of private and public investment very differently (Narayan, 2004; Şen and Kaya, 2014; Mohanty, 2018; Shah et al., 2019).	IMF fiscal affairs department
INFLATION	GDP deflator (% annual)	Healthy and normal level of inflation contributes toward economic development of the country (Dollar and Kraay, 2001).	WDI
IQ	Institutional quality	Institutional quality promotes economic development	Calculated from International Country Risk Guide (ICRG). ICRG (2018) data using the principal component analysis (PCA) methodology

**TABLE 4:** Correlation matrix of six individual institutional indicators.

	BQ	COR	DA	LO	GS	SE
BQ	1.0000					
COR	0.4535	1.0000				
DA	0.3708	0.2902	1.0000			
LO	0.3082	0.3917	−0.0814	1.0000		
GS	−0.0283	0.1966	−0.2488	0.2470	1.0000	
SE	0.5780	0.4537	0.1640	0.5318	0.1372	1.0000

*Authors’ estimation.*

## Empirical Results and Discussion

### Statistical Description of Variables

We have reported our descriptive statistics in Table 5. As per the results of Table 5, the mean value of FDIO (%GDP) is 0.87 and FDII (%GDP) assumes the mean value of 3.33. Descriptive statistics suggest that FDI inflows are much higher in developing countries compared to the level of FDIO. The mean values of PUBI (%GDP) is 10.30 while the average level of PRI (%GDP) is 26.55. The rate of inflation is 11.23% annually.

**TABLE 5:** Descriptive statistics.

Variable	Obs	Mean	Std. Dev.	Min	Max
FDIO	1,220	0.87	2.47	–24.94	22.59
IQ	1,220	0.000	1.57	–3.62	4.90
FDII	1,220	3.33	4.18	–6.05	50.01
FDIO*FDII*IQ	1,220	20.15	158.80	–765.46	2584.40
PRI	1,220	26.55	14.65	0.0072	85.12
PUBI	1,220	10.30	8.87	0.000	63.71
INFLATION	1,220	11.23	79.66	–29.69	2630.12

*Authors’ calculation.*

### Empirical Estimations of Panel Unit Root Tests and Cross-Sectional Dependency Methods

Before applying panel unit test methods, we have to check cross-sectional dependency (CD) in the residuals across the units. Panel unit root tests that consider cross-sectional independence across the units can suffer from lower power or loss power of degree of freedom if applied on the panel dataset that suffers from the issue of cross-sectional dependency (Sadorsky, 2013). We have applied the CD test introduced by Pesaran (2004) in order to check the cross-sectional dependency in the variables of our proposed study. This CD test produces unbiased results as the number of cross-sectional units increases and reaches infinity. Our null hypothesis of the CD test is the cross-sectional independence across the units against the alternative hypothesis of cross-sectional dependence across the variables of our proposed study. As per results, which are reported in Table 2, there is the existence of cross-sectional dependence across the variables of our study. The correlation (ρ)^ matrix of six individual institutional indicators are reported in Table 4. Due to presence of cross-sectional dependency in our dataset, we have applied the CIPS^5^ panel unit root [Z (t-bar)] test that is proposed by Pesaran (2007).

The CIPS test confirms that all variables are stationary at the first difference. These unit root test results are reported in Table 2 and results of the correlation matrix of the six individual institutional indicators are reported in Table 4.

Due to the presence of cross-sectional dependency in the variables of our study, we apply CS-ARDL methods to overcome the issue of endogeneity and cross-sectional dependency.

### The Impact of Foreign Direct Investment Inflow, Foreign Direct Investment Outflows, and Institutional Quality on Domestic Capital Formation in Developing Countries

Empirical estimations in our baseline and extended models show that FDI outflows insignificantly affect DCF in the long run as per result estimations which are reported in Table 6. Among the other variables, FDII and IQ significantly contribute to DCF in the short run and long run as well. Institutional quality significantly reduces the cost of the running businesses and subsequently, positively contributes to the expansion of domestic capital resources. Our result findings are in line with those of Ali et al. (2010), Buchanan et al. (2012), and Vojtovic et al. (2019) which conclude that sound institutional quality safeguards the property rights of the industry and subsequently, this increases the confidence of multinational enterprises to invest abroad and in return increases their domestic reserves by gaining optimum profits by making huge investments abroad in the host country. The interactive effects of FDII, FDIO, and IQ (FDIO × FDII × IQ) show insignificant effects or do not contribute to aggregate domestic capital formation in the long run and these findings are in line with those of Carbonell and Werner (2018) which found insignificant effects of institutional quality and foreign capital flows on DCF in the long run. The sign of the error correction term suggests that there exists a valid relationship between the dependent variable (DCF) and the other variables of interest in our proposed study. The disequilibrium adjustment rate is 36.17, 33.54, and 30.63% in the baseline and extended models, respectively.

**TABLE 6:** The impact of FDII, FDIO, and IQ on DCF.

DV: DCF	M1.1	M1.2	M1.3
**Error correction (EC)**	−0.3617***	−0.3354***	−0.3063***
	(−9.40)	(−9.28)	(−7.44)
**Long-run estimates**			
FDIO	0.0667	0.1237	0.0345
	(0.54)	(0.85)	(0.26)
FDII	0.3170***	0.3013***	0.4017***
	(6.59)	(6.18)	(7.51)
IQ	1.8494***	1.7387***	1.7399***
	(10.00)	(8.02)	(9.34)
FDIO*FDII*IQ		–0.0026	–0.0018
		(−0.47)	(−0.33)
INFLATION			−0.0181***
			(−3.32)
**Short-run estimates**			
Δ FDIO	0.5985*	0.5920	0.8106
	(1.95)	(1.07)	(1.27)
Δ FDII	0.3712***	0.4231***	0.4280***
	(3.41)	(3.38)	(3.18)
Δ IQ	1.2122***	1.2562**	1.1743**
	(2.64)	(2.36)	(2.08)
Δ FDIO*FDII*IQ		–0.1301	0.0772
		(−0.31)	(0.20)
Δ INFLATION			0.0369
			(1.49)
Constant	8.37***	7.61***	5.73***
	(9.46)	(9.31)	(7.72)
Observations	1,159	1,159	1,159
Country	61	61	61

Authors’ estimation. ‘****,” “**,” and “*” denote the level of significance at 1, 5, and 10%, respectively. **DV,** DV denotes dependent variable in our model; IQ stands for institutional quality; PRI denotes private capital formation, PUBI stands for public capital formation; () denote t-values in the parenthesis.

Looking in to the short-run results in Table 6, we found that FDII and IQ positively and significantly affect domestic capital formation. However, the signs of coefficients of FDIO, the variable of interaction effects (FDIO × FDII × IQ), and inflation rate are insignificant and do not contribute to aggregate domestic capital formation in the long run and short run in developing countries which is contrary to expectation. The insignificant and unexpected result in Table 6 indicates to the problem of aggregation bias and demands deep down sectoral, i.e., public and private capital formation, analysis.

### The Impact of Foreign Direct Investment Inflow, Foreign Direct Investment Outflows, and Institutional Quality on Private Capital Formation in the Developing Countries

Following results reported in Table 7, FDI outflows significantly augment PRI in the long run for developing countries. The long-run FDIO coefficient is 0.5348 in the baseline model. Henceforth, we can infer from empirical results that FDIO expands PRI significantly in the long run while in the short run, FDIO does not contribute to PRI. As per empirical results, we conclude that FDIO is an effective tool to augment PRI significantly in the long run for developing countries. Our result estimations conform with those of Fabry (2001) which suggested that FDIO is a good policy tool to stimulate private capital in the long run. Our results suggest that other variables of interest such as FDII and IQ significantly expand PRI in the long run while INFLATION significantly stimulates PRI in the short run and long run as well. As per the results in Table 7, we notice the highly significant incoming impact of FDIO, FDII, and IQ on PRI in our models, once more confirming that FDII, FDIO, and IQ are good policy tools to expand PRI in the home country. Our result findings agree with those of Calderón (2009) which claim that foreign capital flows and sound institutions increase the private capital of the home country in the long run. Although FDIO significantly contributes to PRI in developing countries, the incoming effect of FDII on PRI is much higher than FDIO in our baseline and extended model for developing countries. Henceforth we conclude that compared to FDIO, FDII can quickly reform the developing regions because FDI inflows are higher than FDIO in the developing region. FDIO is not quite as high in developing countries because the local industry is not quite developed and domestic reserves are scarce. Consequently, FDI outflows are lower in developing countries due to poor local industrial structure and scarce domestic capital savings. Moreover, IQ reduces the business transaction costs and expands PRI by stabilizing the economy. Empirical results suggest that one unit increase in IQ enhances PRI by 2.6585 units.

**TABLE 7:** The impact of FDII, FDIO, and IQ on PRI.

DV: PRI	M1.1	M1.2	M1.3
**Error correction (EC)**	−0.1954***	−0.1820***	−0.1106***
	(−4.71)	(−4.27)	(−4.57)
**Long-run estimates**			
FDIO	0.5348***	0.5141**	−2.04***
	(2.69)	(2.04)	(−5.74)
FDII	2.16***	1.88***	2.13***
	(14.62)	(15.44)	(7.14)
IQ	2.65***	2.28***	3.02***
	(7.13)	(5.37)	(3.01)
FDIO*FDII*IQ		0.1513***	0.3828***
		(4.71)	(4.96)
INFLATION			0.3586***
			(3.64)
**Short-run estimates**			
Δ FDIO	0.4307	0.5302	–0.5083
	(0.57)	(0.37)	(−0.34)
Δ FDII	0.2512**	0.0742	0.0375
	(1.96)	(0.34)	(0.15)
Δ IQ	–0.4930	–0.3155	0.2883
	(−0.90)	(−0.58)	(0.46)
Δ FDIO*FDII*IQ		–0.4171	–0.9396
		(−0.47)	(−0.89)
Δ INFLATION			0.1606***
			(2.60)
Constant	2.83***	2.70***	6.96***
	(2.62)	(2.61)	(4.17)
Observations	1,159	1,159	1,159
Country	61	61	61

“****,” “**,” and “*” denote the level of significance at 1, 5, and 10%, respectively. **DV,** DV denotes the dependent variable in our model; IQ stands for institutional quality; PRI denotes private capital formation, PUBI stands for public capital formation; () denote t-values in the parenthesis. Authors’ estimation.

As per the results of our extended models, we notice that joint interaction effects of FDII, FDIO, and IQ (FDIO × FDII × IQ) promote PRI in the long run. There is a strong positive link between FDII, FDIO, and IQ to promote PRI in the long run. Our result findings agree with those of Cao and Jariyapan (2012) which claim that there is good interlinkage between FDII, FDIO, and IQ to promote PRI in the long run. Hence, if a country’s institutional quality is good, foreign direct investment (FDII, FDIO) is expected to promote private capital formation in the long run. Economics endowed with strong institutional quality will attract FDI inflows and FDI outflows, it is highly expected that a high level of FDII and FDIO to these countries will expand private capital formation in the developing countries. Institutional quality is strongly linked with outbound FDI as well as inbound FDI to stimulate private capital formation in the developing countries. The joint interaction effect (FDIO × FDII × IQ) of FDII, FDIO, and IQ is positive in our regression analysis as expected. The value of the coefficient of the ECT (error correction term) in the models suggest that there exists a stronger long-run relation between the dependent variable and other explanatory variables of interest in our model in our proposed study. The value and sign of the ECT coefficient suggest (see Table 7) that economic shocks in the short run will revert back to long-run equilibrium by 19.54, 18.20, and 11.06% in the baseline model and extended models, respectively.

### The Impact of Foreign Direct Investment Inflow, Foreign Direct Investment Outflows, and Institutional Quality on Public Capital Formation in Developing Countries

We explored the impact of FDIO, FDII, IQ, and other variables of interest on PUBI in the long run and short run as well. Our empirical results are reported in Table 8. As per result estimations, we notice that FDIO insignificantly affect PUBI in the developing countries in the long run except in our second extended model. However, in the second extended model, an FDIO increase significantly reduced the public capital formation in developing countries in the long run. Our result estimations are in line with those of Andrei (2012) and Gherghina et al. (2019) which claim that FDIO decreases public capital formation in the long run. FDII contributes positively and significantly to public capital formation in the long run in our baseline and extended models except in the second extended model.

**TABLE 8:** The impact of FDII, FDIO, and IQ on PUBI.

DV: *PUBI*	M1.1	M1.2	M1.3
**Error correction (EC)**	−0.2858***	−0.2733***	−0.2576***
	(−7.96)	(−7.32)	(−6.83)
**Long-run estimates**			
FDIO	−0.0035	−0.0673	−0.0765**
	(−0.06)	(−1.31)	(−2.07)
FDII	0.1420*	0.1357*	−0.0005
	(1.84)	(1.70)	(−0.01)
IQ	−1.18***	−1.25***	−1.55***
	(−6.20)	(−6.95)	(−7.56)
FDIO*FDII*IQ		0.0316**	0.0356***
		(2.37)	(2.82)
INFLATION			−0.0239**
			(−2.29)
**Short-run estimates**			
Δ FDIO	0.1084	0.0198	−0.1028
	(0.26)	(0.02)	(−0.13)
Δ FDII	0.1798	0.1732	0.1772
	(1.44)	(1.06)	(1.11)
Δ IQ	−0.2898	−0.3262	−0.3829
	(−0.86)	(−0.89)	(−1.00)
Δ FDIO*FDII*IQ		1.2927	1.215
		(1.32)	(1.21)
Δ INFLATION			0.0328
			(1.22)
Constant	0.2985	0.1617	0.9013***
	(1.10)	(0.61)	(3.51)
Observations	1,159	1,159	1,159
Country	61	61	61

“****,” “**,” and “*” denote the level of significance at 1, 5, and 10%, respectively. **DV:** DV denotes the dependent variable in our model; IQ stands for institutional quality; PRI denotes private capital formation, PUBI stands for public capital formation; () denote t-values in the parenthesis. Authors’ estimation (UNCTAD, 2004; Al-sadiq, 2013).

As per the results, we notice that IQ significantly decreases PUBI in the long run in our models. In our extended models, the variable of the interaction effects term (FDIO × FDII × IQ) of FDII, FDIO, and IQ significantly expand PUBI in the long run. In our extended models, the interaction effects of FDII, FDIO, and IQ significantly increased PUBI in the long run even though IQ decreases PUBI in the long run individually. IQ has strong linkage with FDIO and FDII to stimulate PUBI in developing countries. Thus, quality institutions with good interlinkage of FDII and FDIO complement PUBI in the long run. Our result estimations conform with those of Andrei (2012) and Gherghina et al. (2019) which claim that institutional quality is strongly linked with FDII and FDIO to promote PUBI in the long run. Henceforth, multinational companies will be hesitant to invest in the regions with corrupt institutional quality even though these countries are financially open to receive international capital inflows. The interaction effect (FDIOxFDIIxIQ) between FDII, FDIO, and IQ is positive in our regression analysis as expected. In our extended models, the interaction effects (FDIOxFDIIxIQ) of FDII, FDIO, and IQ also contribute significantly and positively to public capital formation in the long run. Also, the inflation rate contributes significantly and negatively to PUBI in the long run.

## Conclusion and Recommendations

Our empirical research study analyzes the interaction effects of foreign direct investment and institutional quality in promoting aggregate domestic capital formation in developing countries. Using 61 developing economies over the time Appendix span from 1998 to 2017 (annual data), the paper compares such effects by decomposing DCF into public capital and private capital and this is the innovative aspect of this paper. We have applied CS-ARDL methods for estimations in order to overcome the issues of endogeneity and cross-sectional dependency in our dataset. In fact, understanding the different determinants of public vs. private capital formation is important for economic policy in the developing countries. Our empirical results show that FDI outflows augment private capital formation and additionally, institutional quality also upsurges private capital formation. Conversely, as per results, FDI outflows obstruct public capital formation and IQ crowds out public capital formation significantly while private capital crowds out FDI inflows. As per estimations, we notice that FDIO crowds in private capital formation, thus we conclude that the majority of the sectors are controlled by the private sector for developing countries and the role of the public sector is quite minimal. We conclude that private and public capital possess different attributes; thus, clubbing them together might result in aggregation bias. Our result estimations provide several policy implications.

Our empirical results show that interaction effects (FDIO × FDII × IQ) of FDII, FDIO, and IQ are insignificant on DCF and additionally, FDIO insignificantly affects DCF individually in the long run. However, the interaction effects (FDIO × FDII × IQ) of FDII, FDIO, and IQ report positive and significant results when we decompose DCF into private and public capital formation, and FDIO contributes significantly to private capital formation in the long run. This indicates to the problem of aggregate domestic capital formation bias. The results show that FDIO strongly increases PRI and significantly decreases PUBI in developing countries. These empirical findings show that FDIO has been connected to various enterprises ranging from local business to the global chains of production. The variable of interaction effects (FDIOxFDIIxIQ) term of FDII, FDIO, and IQ is significant and positive in the regression analysis as expected for public and private capital formation. Empirical estimations show that institutions are strongly connected with FDII and FDIO to promote public or private capital formation in the long run. Effective institutions promote FDII and FDIO as well; it is highly expected that FDII and FDIO to these countries will complement private and capital formation in the long run. Conversely, weak and corrupt institutions perhaps will generate a great deal of investment-related risks, and thus in return, multinational companies would be misguided to invest aboard in host countries endowed with corrupt institutions even though these countries liberalize their polices to receive a higher level of international capital flows.

In terms of policy implications, the findings of our study suggest that GDP growth and associated productivity gains encourage firms to make higher cross-border investments. However, since the decline in FDIO will not harm the developing countries either, we suggest that the government needs to find a balanced approach to boost cross-border investment, but still keep it within a moderate range to stimulate FDIO in the long run. These implications are useful for other transition countries that are seeking sustainable policies to boost FDIO.

In terms of the limitations of the study, it is recommended that future research can be extended to advanced or transition economies to gain broader conclusions.

## Data Availability Statement

The data analyzed in this study is subject to the following licenses/restrictions: dataset will be available from corresponding author upon suitable request. Requests to access these datasets should be directed to corresponding author.

## Author Contributions

All authors listed have made a substantial, direct, and intellectual contribution to the work and approved it for publication.

## Conflict of Interest

The authors declare that the research was conducted in the absence of any commercial or financial relationships that could be construed as a potential conflict of interest.

## Publisher’s Note

All claims expressed in this article are solely those of the authors and do not necessarily represent those of their affiliated organizations, or those of the publisher, the editors and the reviewers. Any product that may be evaluated in this article, or claim that may be made by its manufacturer, is not guaranteed or endorsed by the publisher.

## Footnotes

1
https://www.imf.org/en/Publications/Policy-Papers/Issues/2018/12/04/pp120618gcc-trade-and-foreign-investment

2 Learning by doing and learning by watching are the two important channels of FDI spillover impact.

3 Government stability (GS), democratic accountability (DA), law & order (LO), bureaucracy quality (BQ), socio-economic conditions (SE), and corruption (COR).

4 First principal component analysis.

5 Cross-sectionally augmented panel unit root test.

## Appendix

### List of Developing Countries

Algeria, Angola, Argentina, Bahrain, Bahamas, Bangladesh, Bolivia, Brazil, Botswana, Burkina Faso, Cameroon, China, Colombia, Chile, Congo Rep, Costa Rica, Cote d’Ivoire, Dominican Republic, Congo Democratic Republic, Ecuador, El Salvador, Egypt, Gabon, Ghana, Guatemala, Guinea, Indonesia, India, Honduras, Iran, Israel, Kuwait, Korea, Jordan, Kenya, Malawi, Malaysia, Mali, Mexico, Morocco, Namibia, Niger, Nigeria, Pakistan, Panama, Peru, Philippines, Paraguay, Russia, Singapore, Saudi Arabia, Senegal, South Africa, Sri Lanka, Thailand, Turkey, Tunisia, Uruguay, United Arab Emirates, Venezuela, Zimbabwe.
